# Supplementary material for: Direct Photochemical Synthesis of Substituted Benzo[b]fluorenes
Source: Org Lett. 2024 Nov 25;26(48):10364–8. doi: 10.1021/acs.orglett.4c03978 (PMC11629382; doi:10.1021/acs.orglett.4c03978)
Supplement: Supplementary file 2 — ol4c03978_si_002.zip [file ol4c03978_si_002.zip › FID for Publication/20240731_RC-2-C-4-78_01/plots/RC-2-C-4-78_HSQCAD_20240731_01_plot01.pdf]

RC-2-C-4-78

Location **26** | Pulse sequence **HSQCAD** | Temperature **25** | Suboperator **RuairiCrawford**
Date collected **2024-07-31** | Solvent **cdcl3** | Spectrometer **localhost.localdomain-vnmrs500** | Operator **MBaumann**

SAMPLE

date	Jul 31 2024
solvent	cdcl3
sample	20240731_RC-2-C-4-78_01

ACQUISITION

sw	5040.3
at	0.150
np	1512
fb	4000
ss	32
d1	1.000
nt	2

2D ACQUISITION

sw1	21361.8
ni	96
phase	arrayed

PRESATURATION

satmode	n
wet	n

TRANSMITTER

tn	H1
sfrq	499.721
tof	-435.4
tpwr	58
pw	6.300

DECOUPLER

dn	C13
dof	-2485.2
dm	nny
decwave	W40_P7374
dmf	32258
dpwr	43
pwxlvl	62
pwx	14.000

HSQC

j1xh	146.0
nullflg	y
mult	2

FLAGS

hs	nn
sspul	y
PFGflg	y
hsglvl	5736

ADIABATIC

pwx180ad	P7374_ad300
pwx180adR	P7374_ad300R
pwx180	465.4
pwxlvl180	57
pwx180ref	P7374_ref200
pwx180r	2000.2
pwxlvl180r	48

SPECIAL

temp	25.0
gain	42
spin	0

F2 PROCESSING

gf	0.069
gfs	not used
fn	2048

F1 PROCESSING

gf1	0.004
gfs1	not used
proc1	lp
fn1	2048

DISPLAY

sp	-457.0
wp	5040.3
sp1	-1257.5
wp1	21361.8
rfl	457.0
rfp	0
rfl1	1257.5
rfp1	0

PLOT

wc	142.6
sc	0
wc2	127.1
sc2	12.7
vs	363
th	4
ai	cdc ph

ACQUISITION ARRAYS

array	phase
arraydim	192
i	phase
1	1
2	2

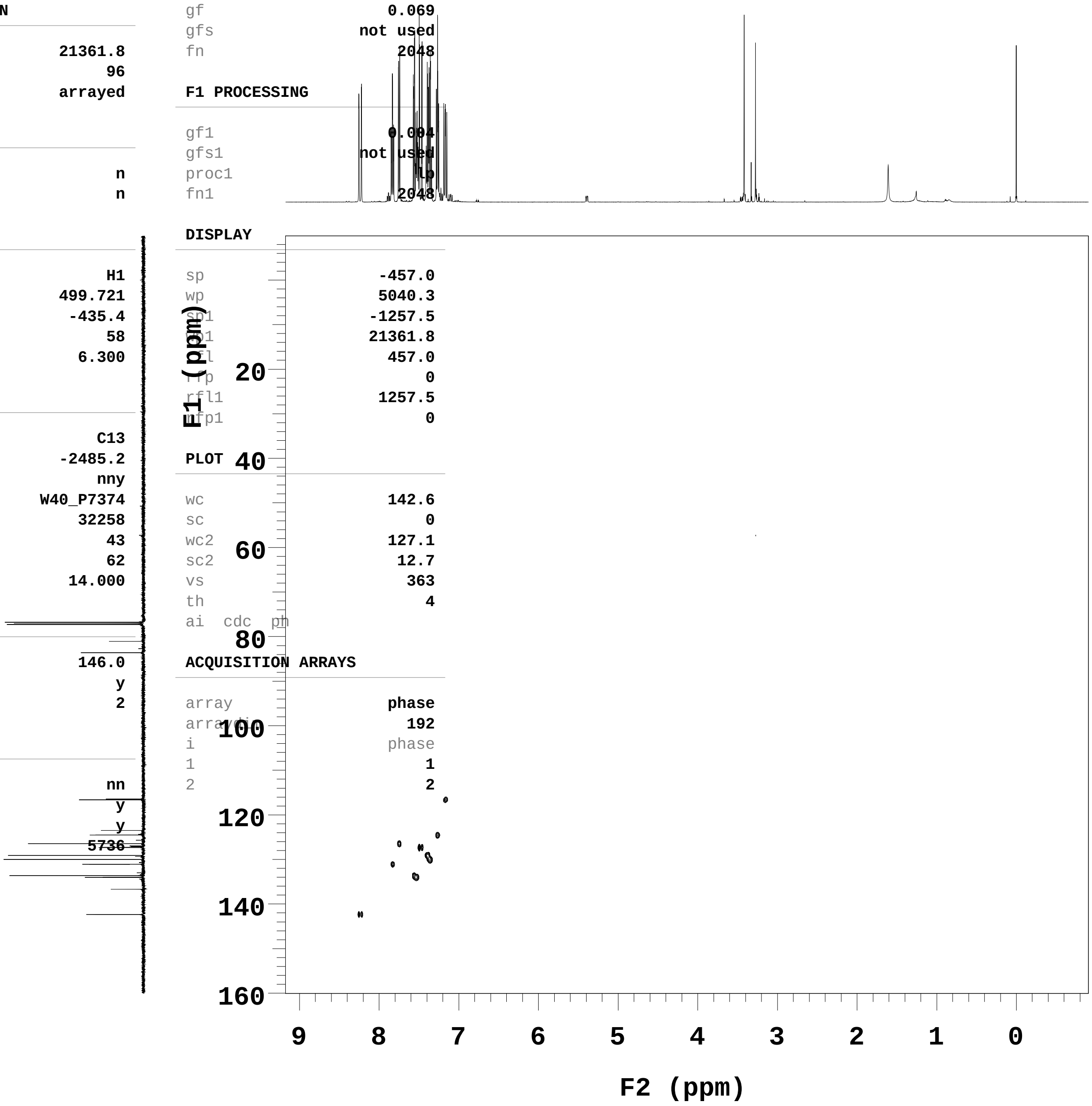

Plotname: RC-2-C-4-78_HSQCAD_20240731_01_plot01

Data file /home/data/MBaumann/cscb500/RuairiCrawford/2024/20240731_RC-2-C-4-78_01/RC-2-C-4-78_HSQCAD_20240731_01.fid Plot date 2024-07-31
